# Poly(Amide-imide) Aerogel Materials Produced via an Ice Templating Process

**DOI:** 10.3390/ma11020233

**Published:** 2018-02-03

**Authors:** Matthew D. Gawryla, Eric M. Arndt, Miguel Sánchez-Soto, David A. Schiraldi

**Affiliations:** 1 Department of Macromolecular Science & Engineering, Case Western Reserve University, Cleveland, OH 44106-7202, USA; mgawryla@hotmail.com (M.D.G.); earndt1@gmail.com (E.M.A.); 2 Centre Catalá del Plástic, Universitat Politécnica de Catalunya. BarcelonaTech, 08022 Terrassa, Spain; m.sanchez-soto@upc.edu

**Keywords:** aerogel, clay, composite, poly(amide-imide), mechanical properties

## Abstract

Low density composites of sodium montmorillonite and poly(amide-imide) polymers have been created using an ice templating method, which serves as an alternative to the often-difficult foaming of high temperature/high performance polymers. The starting polymer was received in the poly(amic acid) form which can be cured using heat, into a water insoluble amide-imide copolymer. The resulting materials have densities in the 0.05 g/cm^3^ range and have excellent mechanical properties. Using a tertiary amine as a processing aid provides for lower viscosity and allows more concentrated polymer solutions to be used. The concentration of the amine relative to the acid groups on the polymer backbone has been found to cause significant difference in the mechanical properties of the dried materials. The synthesis and characterization of low density versions of two poly(amide-imide) polymers and their composites with sodium montmorillonite clay are discussed in the present work.

## 1. Introduction

Polymer aerogel represent a family of low density materials which are typically produced by either supercritical or freeze drying of wet gels [1,2]. The supercritical drying process developed from early work by Kistler [3], who showed that colloidal silica could be generated via a sol-gel process in an alcohol, solvent exchanged with acetone, then dried using supercritical carbon dioxide. Meador [4], Leventis [5] and others have expanded the field to generate polymer-based aerogels in a similar manner. A great advantage of the supercritical drying approach is that the exquisite structure generated in the wet gel state can be preserved in the finished aerogel, producing materials of high surface area and nanoporosity, leading to material with exceptional thermal insulative properties. Downsides to the use of the supercritical process include the handling and removal of large quantities of organic solvents, and use of capital-intensive supercritical carbon dioxide drying. The freeze drying process for producing aerogels produces low density materials whose structures are not dominated by the delicate gel structure of wet gels, but rather materials whose structures are the negatives of the frozen ice structure; polymers and fillers are forced into the grain boundaries of the ice lattice, which is then sublimed away to leave a porous material behind [6]. Because the materials produced using such an ice templating method are coarser than those generated using supercritical drying, they tend to possess lower surface areas, lack nanoporosity, and exhibit typical polymer foam-like thermal conductivities [7]. The freeze drying process benefits from the use of water as solvent and simplicity in the freeze drying process, and is amenable to a wide range of polymers, such as poly(vinyl alcohol) [8] and bio-based alginate [9], casein [10], pectin [11], gelatin [12], natural rubber [13] and hyaluronic acid [14]. These ice-templated polymer aerogels generally benefit from incorporating smectite clays, such as montmorillonite or bentonite, which form exfoliated hydrogels at approximately 2 wt. % in water, and upon freezing form well-dispersed polymer/clay composite materials [15]. Supercritical drying of polymer clay systems, a process which combines elements of both processes described above, has also been reported, and is a method of producing interesting and useful materials [16].

**Figure 1:**
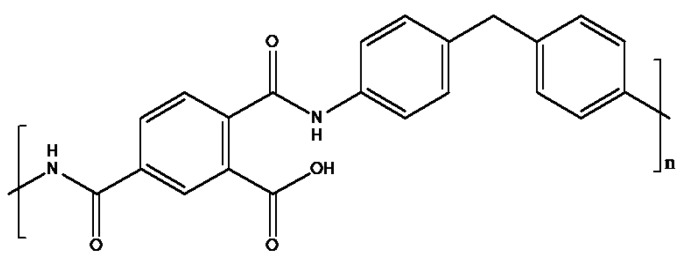
Structure of poly(amide-imide) Torlon^®^ AI-30 (Solvay Advanced Polymers LLC, Alpharetta, GA, USA) in the amic acid form.

Polyimides are high performance polymers often suitable for applications above 200 °C; foaming of polyimides is extremely difficult due to limited melt processing abilities and when melting is possible, low melt viscosities [17]. Many polyimide foams are formed through a powder precursor which expands upon heating, filling a mold of the desired shape [18]. Polyimide foams are primarily developed for aerospace applications which require operating temperatures from cryogenic up to 250 °C [19,20,21,22]. Polyimide aerogels have more recently become of interest, led by the pioneering work of Meador and coworkers at NASA [23,24] and of Leventis [25]. Produced using the supercritical drying process, these polyimide aerogels offer considerable promise in lightweight space/aerospace applications ranging from deacceleration parachutes to antennae. Because polyimides and their starting monomers are typically not soluble in water or other easily sublimable solvents, these aerogels have only been produced using supercritical drying. Poly(amide-imides) are alternatives to polyimides, also possessing high continuous use temperature and good mechanical and chemical properties [26]. Unlike polyimides, poly(amide-imides) are initially produced in a water-soluble poly(amic acid) form, which is then thermally imidized (Figure 1). Such a material then, offers the potential to be converted into a poly(amic acid) aerogel via the freeze drying process, then thermally converted into a finished poly(amide-imide) aerogel. Such a conversion also solves the issue of many aerogels produced via ice templating, that the finished material is latently water soluble; imidized poly(amide-imide) aerogels would not be water soluble, even though they were initially prepared from aqueous solution. A recent work demonstrated that such poly(amide-imide) aerogels can be produced, and that they are highly efficient at removing oil from contaminated waters. In the present work, the structural and mechanical properties of these aerogels, which exhibit excellent thermal, mechanical, and water resistance properties will be described.

## 2. Results and Discussion

**Figure 2:**
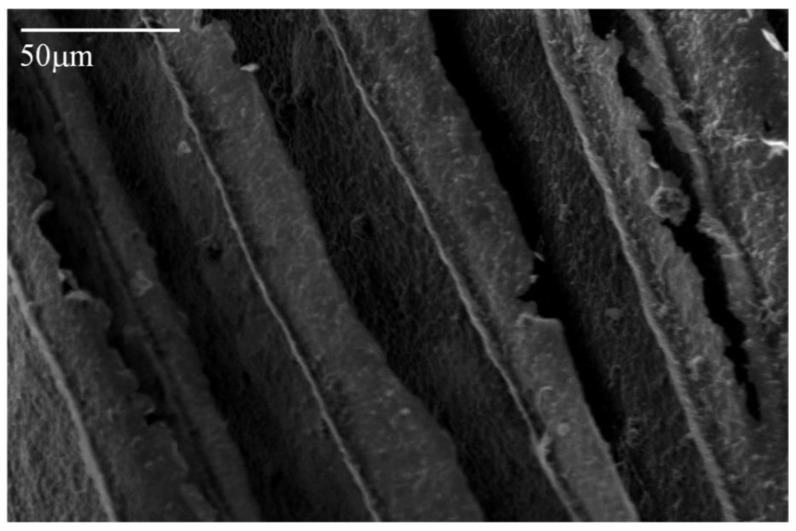
SEM (Scanning Electron Microscope) image of the microstructure of a 5 wt. % Na^+^-MMT/2.5 wt. % (Nanocor Inc. PGW grade, Chicago, IL, USA) AI-30 composite with 2 equivalents of amine.

**Figure 3:**
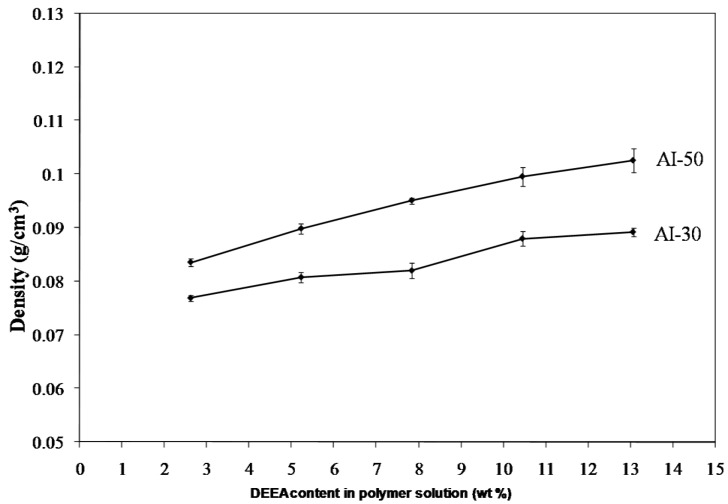
Change in density of 5 wt. % Na^+^-MMT/2.5 wt. % AI-30 composites as a function of amine concentration.

**Figure 4:**
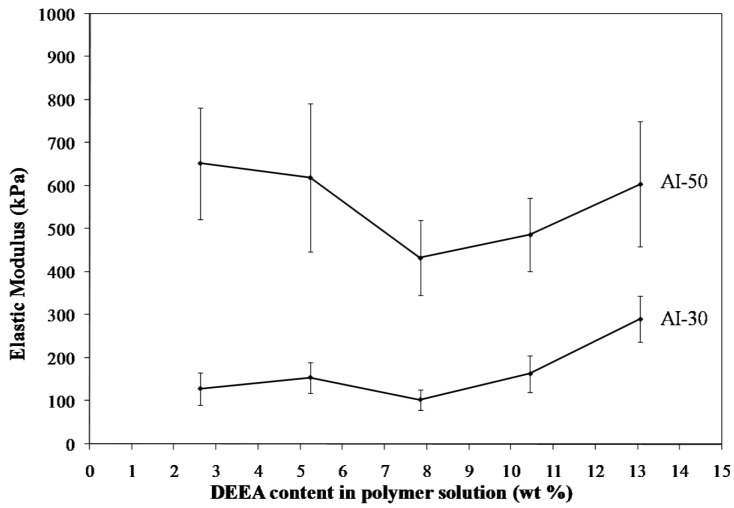
Compressive moduli of 5 wt. % Na^+^-MMT/2.5 wt. % AI-30 composites as a function of amine concentration.

**Figure 5:**
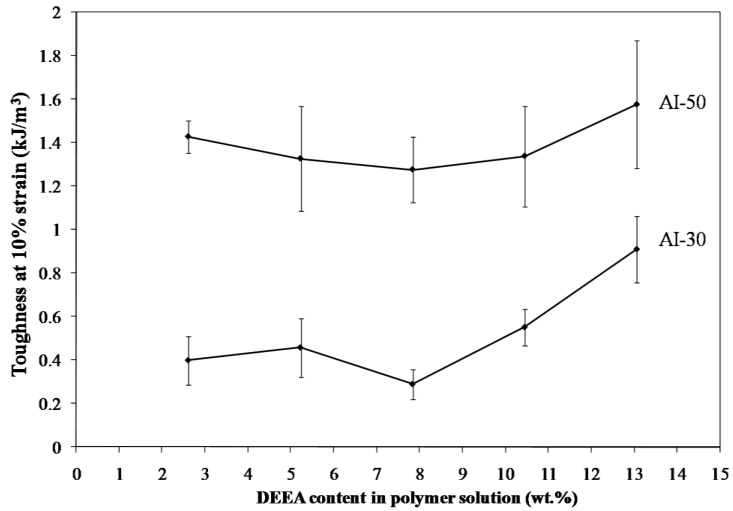
Toughness at 10% strain of 5 wt. % Na^+^-MMT/2.5 wt. % AI-30 composites as a function of amine concentration.

Freeze drying of aqueous solutions of the poly(amic acid) form of the poly(amide-imides) (PAIs) in the presence of diethylaminoethanol (which increases the polymer solubility in water) readily produced mechanically-fragile monoliths, which when thermally treated for several hours at 200 °C under vacuum, produced robust structures with densities in the range of 0.07–0.19 g/cm^3^. The concentration of amine in the preparation solution was found to have little effect on the visual appearance of the PAI clay composites. The composites all exhibited lamellar structures (Figure 2) independent of amine concentration. The structures of freeze dried aerogels have been previously reported, and range from cellular to lamellar solids, depending primarily upon the solute concentrations and freezing temperatures employed [6,14]. Increasing the concentration of amine increased the density of the composites slightly from about 0.085 g/cm^3^ and 0.075 g/cm^3^ to just over 0.1 g/cm^3^ and 0.08 g/cm^3^ for the AI-50 and AI-30 composites respectively (Figure 3). The moduli of AI-50 composites were generally higher than that of AI-30 for all amine concentrations, most likely due to the more rigid *m*-phenylene diamine monomer incorporated into the AI-50 backbone. Within experimental error, the compressive moduli did not change with increasing amine concentration for the AI-50 composite materials (Figure 4). The AI-30 aerogel composites showed a slight increase in modulus at the highest amine concentration; our prior work has shown that mechanical properties increase geometrically with aerogel density, so these increases most likely reflect the modest density increases observed. Toughness, the amount of energy absorbed by a material for a given strain value, were generally independent of the solution amine concentrations (Figure 5). It can generally be concluded then that the amine processing aid used to achieve relatively high poly(amide-imide) solution concentrations had little effect on the final aerogel products.

**Figure 6:**
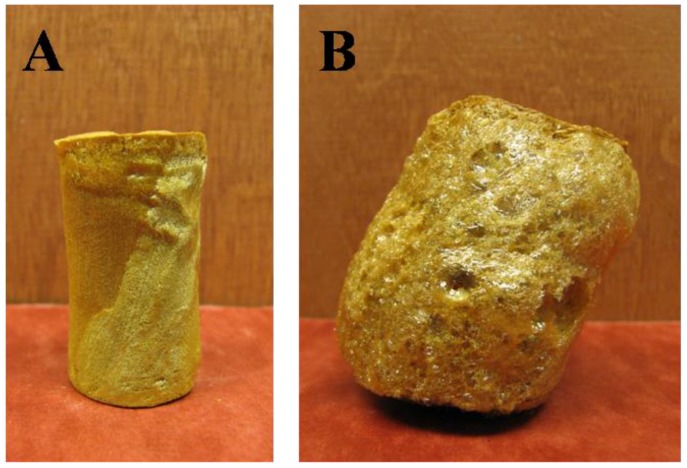
Image of 5 wt. % Torlon^®^ AI-50 materials with (**A**) 6 and (**B**) 10 equivalents of amine.

**Figure 7:**
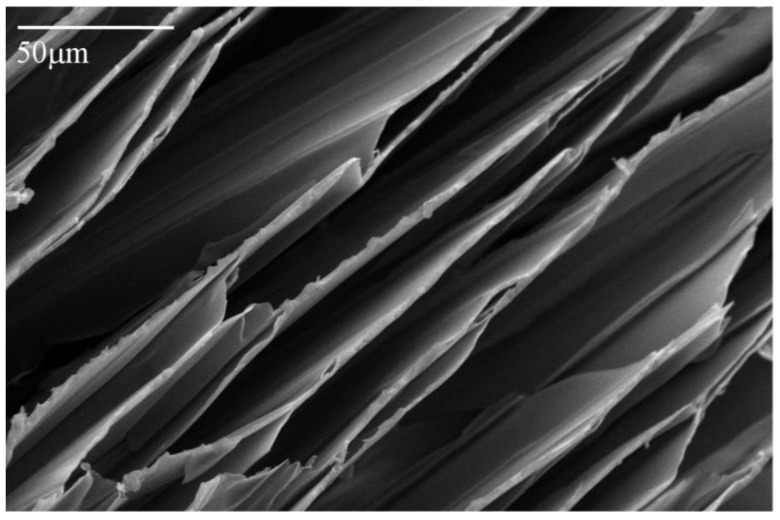
SEM image of the microstructure of a 5 wt. % AI-30 Composite with 2 equivalents of amine.

**Figure 8:**
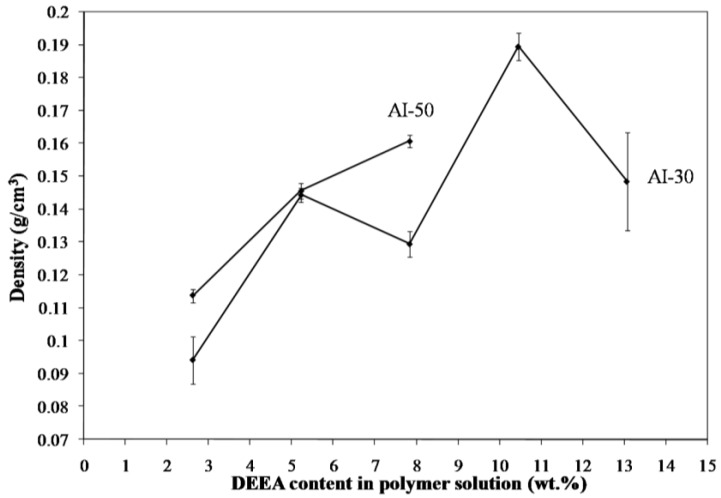
Density as a function of amine content for a 5 wt. % polymer material.

**Figure 9:**
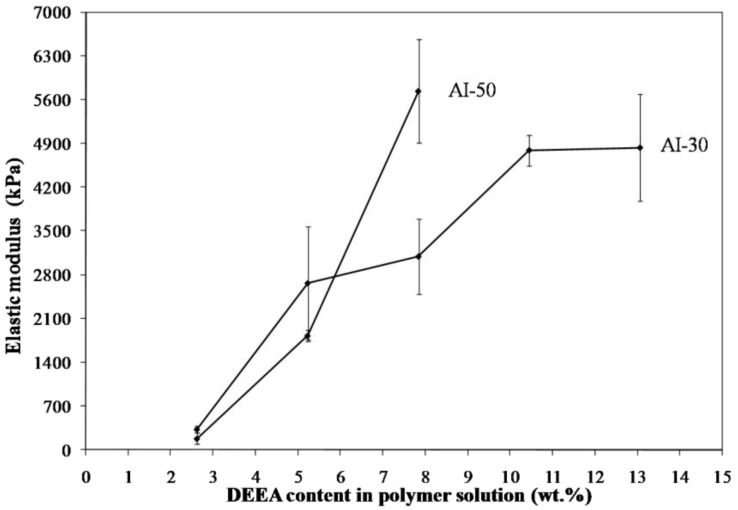
Modulus as a function of amine content for a 5 wt. % polymer material.

**Figure 10:**
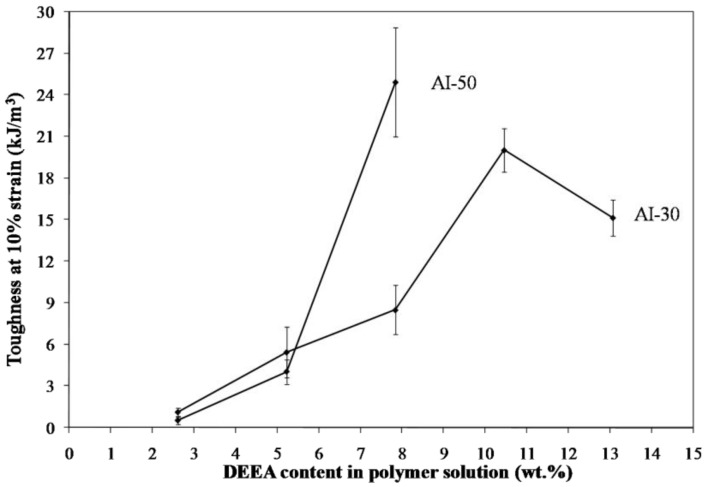
Toughness as a function of amine content for a 5 wt. % polymer material.

Materials containing only polymer, i.e., no clay, were created from the 5 wt. % solutions of AI-30 and AI-50 in order to investigate the effect of clay on the properties. Increasing the amine concentration proved detrimental to the AI-50 composites above six equivalents of amine. The plasticizing effect of such a large excess of amine could contribute to the foam-like structure which is seen in the higher amine concentrations (Figure 6). The foam-like structures were very brittle and unable to be mechanically tested or measured for density due to the non-uniform shape. AI-30 materials did not show the same level of bubble formation and therefore useful materials can be created up to at least 10 equivalents of amine. The internal structure of the polymer-only materials is much smoother and more uniform than that seen when clay is included (Figure 7). When it was included the clay is the main component of the system, 66 wt. % in the final, dried material, and therefore the polymer acts primarily as a binder to the clay particles without the clay in the system, the polymer is free to form smooth layers and in the case of AI-30 these layers are highly flexible due to their dimensions. The density of these polymer-only materials increases with amine content, again due to the plasticized polymer being able to shrink more easily (Figure 8); densities of these aerogels show greater variability than those of typical freeze-dried materials [15] because of the complex interactions of plasticization and crosslinking, effects not studied in detail to date in such systems. With variability in densities comes some variability in modulus, as this mechanical property is highly sensitive to polymer density. As the amine content was increased, the modulus and toughness also showed a general increasing trend (Figure 9 and Figure 10). AI-30 and AI-50 exhibited drastic increases as the amine content increases, gaining over 10 times the stiffness.

As produced via the freeze-drying process, the poly(amic acid) form of the poly(amide-imide) aerogels was, not surprisingly, generally sensitive to moisture. When these structures were placed in a container of water, they of course floated initially, but ultimately dissolved back into solutions similar to those used to produce the frozen starting materials. Upon curing at 200 °C, a remarkable change in this property occurred, and the previously hydrophilic poly(amic acid) aerogels became thoroughly hydrophobic poly(amide-imide) materials. Exposed to water for up to a year, the aerogels were essentially unaffected. Thus, it is possible to produce a hydrophobic, water-stable, low density “foam” from aqueous solution.

**Figure 11:**
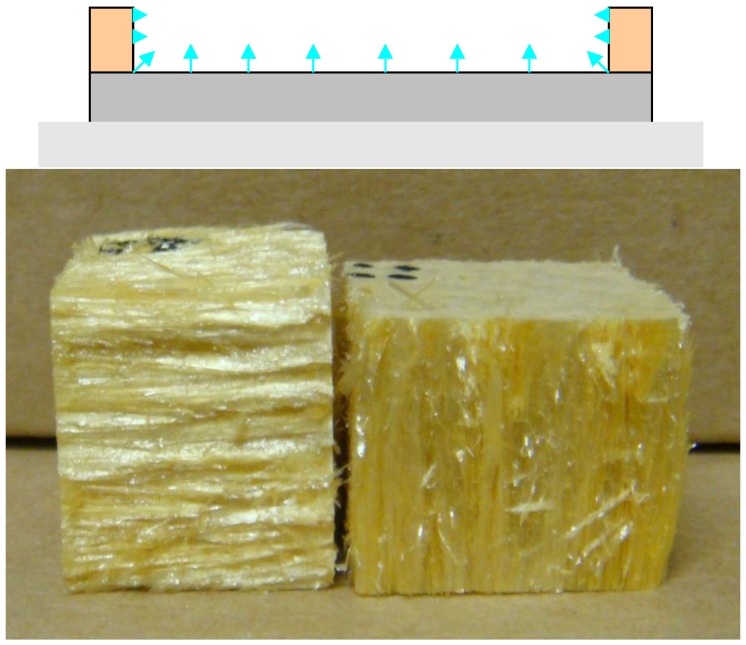
Mold and aerogel monoliths from anisotropic freezing.

Anisotropic poly(amide-imides) were produced by freeze drying the aqueous precursor solutions in the mixed plastic/metal mold. The difference in thermal conductivity of the metal and polymeric sides of the mold led to preferential formation of seed crystals on the metal surface, which when followed by lamellar growth of ice crystals gave rise to anisotropic structures and mechanical properties (Figure 11 and Table 1. As can be seen from the moduli in Table 2, a 50-fold difference in moduli was typical when comparing the ice growth and transverse directions for these structures. With such anisotropy in mechanical properties, it is possible to produce a stiff, high temperature stable, insulating material which in its perpendicular direction is flexible and can be bent around structures, such as pipes and motor housings.

## 3. Materials and Methods

### 3.1. Materials

Sodium Montmorillonite (Na^+^-MMT) (Nanocor Inc. PGW grade, Chicago, IL, USA), and Torlon^®^ AI-30, Torlon^®^ AI-50 (Solvay Advanced Polymers LLC, Alpharetta, GA, USA), diethylaminoethanol (DEAE) (99.5%, Sigma Aldrich, St. Louis MO, USA) were used as received. Deionized water was prepared using a Barnstead RoPure reverse osmosis system (RoPure, Barnsted, Van Nuys, CA, USA). The Torlon^®^ AI-30 chemical structure is given in Figure 1; the AI-50 product differs from AI-30 in that the methylene dianiline monomer is replaced with at 0.7/0.3 mixture of 4,4′-oxydianiline/*m*-phenylene diamine [27].

### 3.2. Clay/Torlon^®^ Composites

Aqueous solutions of Torlon^®^ AI-30 and Torlon^®^ AI-50, were prepared by mixing a calculated amount of diethylaminoethanol and deionized water (Table 2. Values for the moisture content and acid number of the Torlon^®^ AI-30 and Torlon^®^ AI-50 powders were assumed to equal 65 wt. % and 125 mg KOH/g respectively, as per the manufacturer’s literature [28]. The amine solution was then heated to 95 °C and the polymer slowly added. 10 wt. % clay gels were prepared from Na^+^-MMT and deionized water using a model MC2 (Waring, Stamford, CT, USA) mini laboratory blender on high speed. 50 mL of the polymer solution was then slowly added to 55 g of the clay gel while stirring. After mixing, the samples were placed in 18 mL polystyrene vials and frozen in a solid carbon dioxide/ethanol bath. Freeze drying was carried out using a Advantage Model EL-85 (Virtis, Warminster, PA, USA) lyophilizer with an initial shelf temperature of −10 °C, which increases to 25 °C after full vacuum is attained. 5 wt. % polymer solutions were also frozen in the same manner to obtain clay free materials, made entirely of poly(amide-imide). After freeze drying, all samples were removed from the vials and cured in a vacuum oven at 200 °C under vacuum for several hours to convert the poly(amide amic acid) to poly(amide imide) and to remove DEAE from the samples. In the case of directionally-frozen, anisotropic aerogels, a complex mold composed of an aluminum bottom and polystyrene sides was used. As the freezing solution in mold was lowered into the low temperature batch, ice crystals first nucleated on the metal surface, giving rise to largely unidirectional lamellar ice crystallization.

### 3.3. Characterization

Compression samples were cut from the dried monoliths using a band saw such that the sample height was less than or equal to the width (approximately 2 cm). Compression tests were carried out using an model 5565 (Instron, Norwood, MA, USA) universal testing machine with a crosshead speed of 1mm per minute. Stress strain curves were plotted, and the moduli were measured from the initial linear portions of the curves. Densities were calculated from the mass of each sample and their dimensions. Scanning electron microscopy imaging was performed on a XL-30 ESEM (Phillips, Amsterdam, The Netherlands).

## 4. Conclusions

Using the high temperature poly(amide-imide) polymers Torlon^®^ AI-30 and AI-50, low density foam-like materials can be created which exhibit useful mechanical properties. Increasing the amount of amine counter ion in the polymer solution has a larger effect when there is no clay in the material. The clay stabilizes the material and provides a support structure as the amine is being removed during curing. The higher amine concentrations appear to plasticize the polymer and therefore when creating polymer-only materials an upper limit exists.

## Figures and Tables

**Table 1:** Anisotropic PAI aerogel compressive moduli.

	Horizontally Aligned, First Compression Cycle	Horizontally Aligned, Second Compression Cycle	Vertically Aligned, First Compression Cycle	Vertically Aligned, Second Compression Cycle
Compressive moduli, KPa	71 ± 17	67 ± 15	4500 ± 370	3200 ± 390

**Table 2:** Poly(amide-imide) solution compositions.

Stoichiometric Multiplier	Deionized Water (g)	DEAE (g)	Polymer Powder (g)
2	83.1	2.611	14.286
4	80.5	5.222	14.286
6	77.9	7.833	14.286
8	75.3	10.444	14.286
10	72.7	13.055	14.286
